# Surgery versus conservative management for severe pectus excavatum (RESTORE): protocol for a multicentre, randomised, controlled superiority trial

**DOI:** 10.1136/bmjopen-2025-113818

**Published:** 2025-12-24

**Authors:** Rebecca Maier, Joel Dunning, James Wason, Thomas Chadwick, Andrew Bryant, Cristina Fernandez-Garcia, Luke Vale, Gerard R Danjoux, Gillian Wallace, Alistair Levett-Renton, Babu Naidu, Claire Pryor, Peter McCulloch, Rebecca Thursfield, Jonathan Wyllie, Lisa Chang, Leanne Marsay, Enoch Akowuah

**Affiliations:** 1 Academic Cardiovascular Unit, South Tees Hospitals NHS Foundation Trust, Middlesbrough, UK; 2 Population Health Sciences Institute, Newcastle University, Newcastle upon Tyne, UK; 3 South Tees Hospitals NHS Foundation Trust, Middlesbrough, UK; 4 Global Health Economics Centre, London School of Hygiene & Tropical Medicine, London, UK; 5 North Yorkshire Academic Alliance of Perioperative Medicine, York, UK; 6 North Tees and Hartlepool NHS Foundation Trust, Hartlepool, UK; 7 University of Birmingham, Birmingham, UK; 8 PPI Representative, Salford, UK; 9 University of Oxford, Oxford, UK; 10 Alder Hey Children’s NHS Foundation Trust, Liverpool, UK; 11 Newcastle University Translational and Clinical Research Institute, Newcastle University, Newcastle upon Tyne, UK

**Keywords:** SURGERY, Thoracic surgery, Clinical Trial

## Abstract

**Introduction:**

Severe pectus excavatum (PE) may impair cardiopulmonary and physical function. The effectiveness of surgical treatment to correct PE and restore physical function is widely debated due to a lack of high-quality comparative evidence. The RESTORE trial aims to determine the clinical and cost-effectiveness of corrective surgery for severe PE compared with conservative management for the first time in a randomised controlled trial (RCT).

**Methods and analysis:**

RESTORE is a pragmatic, multicentre, RCT with an embedded observational cohort. 200 participants aged ≥12 years with severe PE will be recruited at around 12 National Health Service cardiothoracic surgical centres in England. Participants will be randomised 1:1 to receive either surgery within 3 months of randomisation (intervention arm) or no surgery until after the primary outcome measurement at 1 year (comparator arm). The primary outcome is change in physical functioning from baseline to 1 year as measured by the Short Form Health Survey (SF-36v2) physical function score. The primary economic outcome is cost-effectiveness. The key secondary outcome is change in % predicted VO_2peak_ at 1 year measured by cardiopulmonary exercise test (CPET). Outcomes will be assessed at 1 year post-randomisation in the comparator arm and 1 year post-surgery in the intervention arm. The primary analyses will be undertaken on an intention-to-treat population using a linear mixed-effects model, adjusted for stratification variables via a binary covariate. Other secondary outcomes will include change from baseline of cardiopulmonary function (CPET and spirometry), health-related quality of life using the EuroQol 5 Dimension 5 Level (EQ-5D-5L) and SF-36v2 questionnaires, Hospital Anxiety and Depression Scale and disease specific symptoms (Phoenix Comprehensive Assessment for Pectus Excavatum Symptoms and Pectus Excavatum Evaluation Questionnaire). Adverse events, complications from surgery and operative technical success (Haller and Compression Indices from preoperative and postoperative CT scans) will also be assessed. Health economic analysis will estimate the incremental cost per quality adjusted life year at 1 year.

**Ethics and dissemination:**

The trial was approved by East of Scotland Research and Ethics Service (24/ES/0034). Participants who are ≥16 years of age will be required to provide written informed consent. For participants <16 years of age who are not judged to be Gillick competent, written assent and written informed consent from a parent/guardian will be required. Results will be submitted for publication in peer-reviewed journals and shared with participants, clinicians and commissioners.

**Trial registration number:**

ISRCTN11359779.

**Strengths and limitations of this study**

- This is a large, multicentre, randomised controlled trial to compare corrective surgery versus conservative management in participants with severe pectus excavatum.
- The primary outcome assesses the impact of surgery on physical function, with secondary outcomes assessing quality of life and disease-specific symptom burden.
- The trial will determine the impact of surgery on cardiopulmonary function using cardiopulmonary exercise tests performed using a uniform protocol in all participants.
- The trial will assess both clinical and cost-effectiveness.
- Blinding of participants is not feasible; therefore, to accompany the self-reported outcomes, objective measures of cardiopulmonary function are included.

## Introduction

 Pectus excavatum (PE) is a congenital condition found in between 1 in 400 and 1 in 1000 people.^1^ It can occur at, or soon after birth, although for most people, it occurs during the pubertal rapid growth phase. In PE, the sternum can be pushed towards the spine, narrowing the space in the chest for the heart and lungs. In many cases, PE does not impact health, and many patients who present to physicians are concerned about the psychological impact resulting from the deformity of the chest wall. However, for people with severe PE, narrowing of the chest cavity may limit cardiopulmonary function by causing pulmonary and cardiac compression. This may be, in theory, from restricted right ventricular filling, which results in a fixed stroke volume and cardiac output, especially during exercise.^2^,^6^ Affected individuals may not feel symptoms at rest but develop symptoms, typically dyspnoea, but also tachycardia, syncope, dizziness and pain when they participate in exercise.^7^,^16^ These symptoms may impair physical function (PF) and reduce health-related quality of life.

To treat these symptoms, surgery for PE is often proposed. The procedure lifts the sternum away from the heart, allowing increased cardiac output during exertion and improving cardiopulmonary function.

The effectiveness of surgery to achieve this, however, is not proven and is frequently debated. Many clinicians, commissioners and payers for healthcare believe that the benefits from surgery are restricted to those that are cosmetic and psychological only. For example, in 2019, the National Health Service (NHS) in England decommissioned surgery for PE in England, citing the lack of high-quality comparative data to demonstrate an improvement in physical or cardiopulmonary function. Surgery has subsequently been funded again in England but is restricted to the most severe cases.^17^ Similar restrictions are present in the USA.^18^

Complication rates after Nuss surgery increase with age, with complications seen in around 16% of all cases, and complications requiring reintervention (Clavien-Dindo classification ≥III) accounting for 70% of all complications.^19^ The use of multiple corrective metal bars used to lift and fix the sternum has improved the effectiveness and safety but has significantly increased the cost.

There is, therefore, an urgent need for high-quality evidence to inform guidelines, practice, future commissioning decisions and discussions of risk and benefit with patients for surgical correction of PE. A randomised trial of surgery versus no treatment to RESTORE cardiopulmonary function in severe pectus excavatum (The RESTORE Trial) aims to address the comparative evidence gap by evaluating whether corrective surgery improves PF, cardiopulmonary performance, quality of life, and to assess its cost-effectiveness compared with conservative treatment.

## Methods and analysis

### Study design

RESTORE is a multicentre, superiority, randomised controlled trial (RCT) of corrective surgery for PE (intervention) versus no surgery (comparator). The trial will answer the question ‘Is corrective pectus surgery superior to no surgery as measured by change in SF-36v2 physical function score at 1 year?’

### Population and setting

200 people aged ≥12 with severe PE (Haller index >3.25)^17^ and impaired PF (defined as Short Form Health Survey (SF-36v2) PF score of ≤80),^20 21^ plus physical symptoms attributable to PE will be recruited at around 12 NHS cardiothoracic surgical centres in England. Feasibility to complete all trial-related assessments and surgical expertise in corrective pectus surgery will be confirmed at all centres. Potential participants will be identified from outpatient clinics at participating hospitals, from new referrals, and from wide and targeted advertising of the study using social media and our established patient groups (Pectus support UK Facebook group and the Pectus Matters charity group) to ensure that patients with severe PE are given the opportunity to take part. The recruitment period is from 1 August 2024 to 31 August 2026. The first participant was recruited on 5 August 2024. The primary analysis will be performed once all 1 year data are collected. The overall trial end date is 31 May 2030.

### Eligibility criteria

#### Inclusion Criteria

1. ≥12 years old.
2. A PE deformity with a Haller Index of >3.25, as measured by the internal width of the chest measured at the widest point divided by the distance from the back of the sternum to the anterior vertebral body at its minimum point on CT scan.
3. An SF-36v2 physical functioning score ≤80.
4. The participant must satisfy at least one of the following symptomatic criteria:
  - Significant level of shortness of breath or exercise ability perceived to be below that of their peers (eg, limited by vigorous activities such as running or lifting heavy objects).
  - Presyncope or syncope on exercise.
  - History of arrhythmias that may be due to the pectus abnormality.
  - Dysphagia or swallowing abnormalities in the absence of any other cause.
5. Provide informed consent/assent.
6. Fit to undergo surgery.

#### Exclusion criteria

1. Patients not fulfilling the inclusion criteria.
2. Symptoms relating to causes other than PE.
3. Received previous corrective surgery for PE (Nuss/Ravitch).
4. Unwilling to have surgery for PE.

### Randomisation

Eligible participants will be randomised (1:1) to surgery within 3 months (intervention arm) or no surgery until after the primary outcome measure at 1 year (comparator arm). Randomisation will be completed using a secure, web-based system stratified by surgical technique (Ravitch or Nuss), age (under 16 years old or 16 years old and over) and cardiopulmonary exercise test (CPET) results (≤85% predicted VO_2peak_ mg/mL/kg (or a suboptimal test), or, over 85% predicted VO_2peak_ mg/mL/kg).

### Blinding

The trial is not blinded and participants and clinical teams will be aware of the allocation. A full statistical analysis plan (SAP) will be developed and agreed with an Independent Data Monitoring and Ethics Committee (IDMEC) and Trial Steering Committee (TSC) prior to any unblinded data being provided to the statistician(s) or any analysis by trial arm being undertaken.

### Intervention arm

The RESTORE trial is pragmatic and the choice of surgical procedure (Ravitch/Nuss) will depend on the surgeon and the participant. Participants will be followed for up to 3 years after surgery. Participants in the intervention arm will receive surgery within 3 months of randomisation.

#### Nuss

Nuss is a minimally invasive approach, in which metal bars are used to elevate the sternum. One or more bars may be used. The bar(s) remains in situ for between 2.5 and 3 years postsurgery, after which, they are surgically removed.

#### Ravitch

Ravitch will be used in difficult long-standing cases, where the chest wall is relatively fixed. It involves resection of costal cartilage and lifting of the sternum. The ribs usually require lateral rib fractures and sternal plating with methods such as the elastic stable repair with rib plates.

### Comparator arm

Participants in the comparator arm will not receive surgery until after the primary outcome measurement has been completed 1 year following randomisation.

### Trial procedures

**Figure 1:**
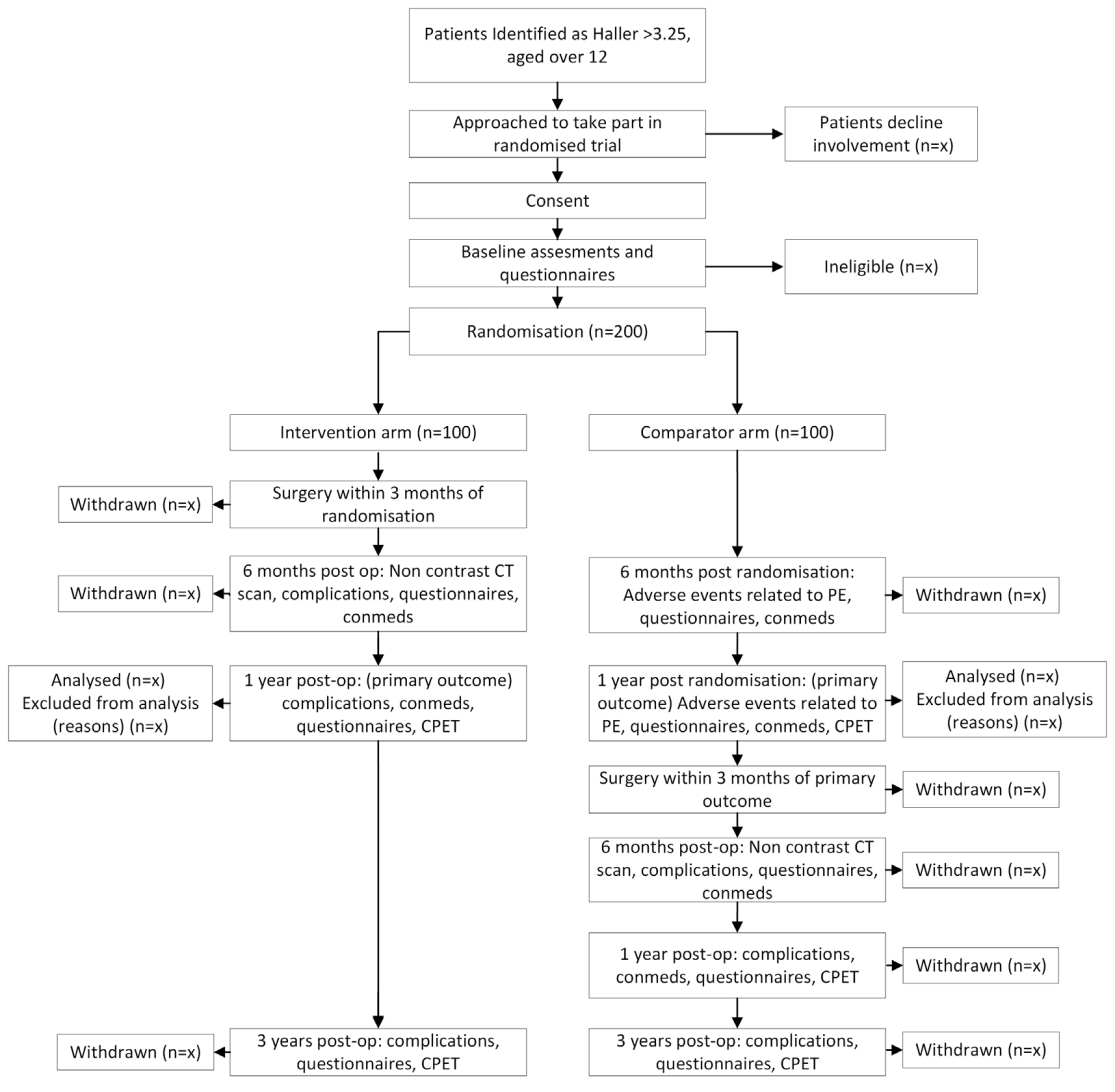
RESTORE trial flow diagram. CPET, cardiopulmonary exercise test; PE, pectus excavatum.

The trial flow diagram is provided in figure 1, and the schedule of events is provided in table 1.

**Table 1:** RESTORE trial schedule of events

	Both arms	Comparator arm only		Both arms				
	Baseline	6 months post-randomisation	1-year post-randomisation	Index surgery	6 months post-op	1-year post-op	3-year post Nuss	6 months after bar removal
Consent	X							
Eligibility	X							
Randomisation	X							
Surgery				X				
Nuss bar removal							X	
Demography	X							
Medical history*	X							
Medications	X	X	X		X	X		X
CT scan	X†				X‡			
Echo/cardiac MRI	X†							
Lung function	X†							
CPET	X§		X¶			X		X
SF-36v2	X	X	X		X	X		X
EQ-5D-5L	X	X	X		X	X		X
PEEQ	X	X	X		X	X		X
PCAPES	X	X	X		X	X		X
HADS	X	X	X		X	X		X
BIDQ	X	X	X		X	X		X
SUQ	X	X	X		X	X		X
DCE			X**			X**		
TTQ		X			X††			
GPAQ	X		X			X		X
Adverse events		X	X	X	X	X		X

* Including previous mental health service use.

† Data collected from standard of care (SOC) assessments, these may have been performed up to 1 year prior to consent except echo/cardiac MRI, which may be performed any time prior to consent.

‡ Postoperative SOC low-dose CT scan may be performed from day 1 up to 6 months post-index surgery.

§ Cardiopulmonary exercise test (CPET) data collected from SOC assessment if ≥16 years of age and performed within 1 year of consent or if <16 years and within 6 months of consent. Repeated as a study assessment if these criteria are not met.

¶ May be performed from 9 months post-randomisation.

** Discrete choice experiment (DCE) to be performed around month 18 after surgery (intervention arm) or after randomisation (comparator).

†† TTQ at 6 months post op is only for intervention arm and is not repeated for the comparator arm.

BIDQ, Body Image Disturbance Questionnaire; EQ-5D-5L, EuroQol 5 Dimension 5 Level; GPAQ, Global Physical Activity Questionnaire; HADS, Hospital Anxiety and Depression Scale; PCAPES, Phoenix Comprehensive Assessment of Pectus Excavatum Symptoms; PEEQ, Pectus Excavatum Evaluation Questionnaire; SF-36v2, Short Form Health Survey; SUQ, Service Use Questionnaire; TTQ, Time and Travel Questionnaire.

### Questionnaires

Questionnaires will be completed by participants directly in the trial database using the CASTOR ePROM facility, or completed on paper or over the telephone, according to their preference.

Physical functioning and overall quality of life will be assessed using SF-36v2,^22^ EQ-5D-5L^23^ and Hospital Anxiety and Depression Scale (HADS).^24^ The symptoms of PE, their impact on quality of life and self-image will be assessed using a modified Pectus Excavatum Evaluation Questionnaire (PEEQ),^25^ Phoenix Comprehensive Assessment of Pectus Excavatum Symptoms (PCAPES)^26^ and Body Image Disturbance Questionnaire (BIDQ).^27^ These questionnaires will be completed at all visits (before randomisation for all participants, 6 months and 1 year following randomisation (comparator arm only) and 6 months, 1 year and 3 years following index surgery (both arms)).

The Global Physical Activity Questionnaire (GPAQ)^28^ is used to collect information on day-to-day levels of physical activity. The GPAQ will be completed at the same time-points as CPET assessments (prior to randomisation, 1 year post-randomisation (comparator arm only) and 1 year and 3 years following index surgery for all participants).

Trial-specific questionnaires for healthcare service use (Service Use Questionnaire (SUQ)), travel time and costs, concomitant medications and medical history have been developed.

SUQ and concomitant medications will be collected at all visits, and medical history will be collected at baseline only. The time and travel costs questionnaire will be collected at 6 months following randomisation (comparator arm) or 6 months following surgery (intervention arm).

### Echocardiogram/cardiac MRI

Cardiac function and the ruling out of cardiac causes for the symptoms of shortness of breath is assessed by transthoracic echocardiogram/cardiac MRI at baseline as standard of care for PE prior to randomisation. Left ventricular ejection fraction and visualisation of valves and other structures of the heart are mandatory.

### CT scan

CT scan measurements are performed at baseline as standard of care for PE to confirm severity as measured by Haller index and within 6 months after index surgery to assess technical success of the operation. The following indices: Haller, Correction, Vertebral, Depression, Asymmetry, Cardiac Compression, Cardiac Asymmetry and Titanic will be measured on inspiration and/or expiration using a non-contrast and low-dose CT scan. The methods for calculating the CT measurements are provided as online supplemental information A.

### Cardiopulmonary exercise test

CPETs will be performed at baseline, 1 year following randomisation (comparator arm), and 1 year and 3 years following index surgery for all participants. The same testing centre should be used for each participant in order to minimise variability. Suboptimal tests where peak respiratory exchange ratio is not >1.1 and peak heart rate (HR) has not reached >85% of that predicted will be repeated once only. Suboptimal data will still be collected and analysed. All tests will be by cycle ergometry consisting of a rest phase, unloaded warm-up, ramp and recovery phases. Predicted peak values for oxygen consumption (VO_2peak_), O_2_ pulse, HR, minute ventilation and power output as well as the predicted ramp will be calculated using the Study of Health in Pomerania (SHIP) calculations for adults^29^ and Blanchard calculations for participants under 16 years of age.^30^ Further details are provided in online supplemental information B.

### Lung function tests

Spirometry will be carried out at baseline as part of standard of care for investigating PE with Forced Expiratory Volume in 1 s (FEV1) and Forced Vital Capacity (FVC) recorded. Where possible, Transfer Factor for Carbon Monoxide, Carbon Monoxide Transfer Coefficient and Alveolar Volume (VA) single breath will be measured at baseline. FEV1 and FVC will be measured during CPET assessments at 1 year and 3 year follow-up visits.

### Outcomes

#### Primary and key secondary outcomes

The primary outcome is change in SF-36v2 PF score between baseline and 1 year, using a 4-week recall period. The key secondary outcome is cardiopulmonary function change between baseline and 1 year, assessed by percentage predicted VO_2peak_ on CPET.

The primary economic outcome is cost effectiveness measured in terms of incremental cost per quality-adjusted life year (QALY) gained over 1 year following index surgery.

#### Additional secondary outcomes

- Measures of cardiopulmonary function by CPET and spirometry at 1 year and 3 years (listed in online supplemental information B).*
- Quality of life measures, including those that understand the impact on mental well-being (EQ-5D-5L and SF-36v2 mental component scores), at 1 year and 3 years.*
- Anxiety and depression scores measured by HADS at 1 year and 3 years.
- Symptoms measured by modified PEEQ and PCAPES questionnaires at 1 year and 3 years.*
- Body image measured by BIDQ at 1 year and 3 years.*
- Measures of technical operative success by Haller Index and Correction index postoperatively.
- Need for revision surgery (complications including the need for unplanned redo surgery and syncope) to 1 year and 3 years.*
- Adverse events of special interest (related to PE not the intervention) to 1 year and 3 years.*
- Major surgical complications to 1 year and 3 years.*
- Cost per participant including interventions costs, costs incurred by the healthcare and social care service and out of pocket expenses for participants and their families/carers at 1 year post-intervention and over the participants’ lifetime.
- Average QALYs per participant estimated from EQ-5D-5L and Short Form 6 Dimensions (SF-6D, derived from SF-36v2) over 1 year.
- Modelled costs and QALYs over the participant’s lifetime.
- Modelled incremental cost per QALY gained over the participant’s lifetime.
- Incremental net benefit of the intervention.
- Participants’ willingness to pay (WTP) for each intervention.

*The 3 year measures will be undertaken >6 months following removal of Nuss bars for those receiving this operation.

### Sample size

35 people with severe PE who had not received surgery were asked to complete the SF-36v2 questionnaire to determine baseline PF. This revealed an SD of 27.4. Assuming Alpha of 5%, a sample size of 170 participants provides 90% power to detect a 15-point difference in PF with a conservative SD=30. This level of expected variance is similar to that found in the National Institute for Health and Care Research UK Mini-Mitral trial.^31^ The published minimal clinically important differences for SF-36v2 PF, including in 12–17 year olds, are 10.^20 21^ Recruitment of 200 participants allows for 15% attrition.

### Statistical analysis

Analysis of the primary outcome of change from baseline in SF-36v2 PF score at 1 year (1 year post-randomisation in the comparator arm, 1 year postsurgery in the intervention arm) will be undertaken on an intention-to-treat (ITT) population using a linear mixed-effect regression model, adjusting for baseline value, site and randomisation stratification variables. The linear mixed-effect model will account for both intra-participant and intra-site correlations using a nested covariance matrix to obtain robust SE to minimise the likelihood of a false conclusion.

We will determine whether corrective pectus surgery is superior to no surgery as measured by a change in cardiopulmonary function assessed by percentage predicted VO_2peak_ on CPET. This is measured at baseline, and 1 year after randomisation (comparator arm) and 1 year after surgery (intervention arm).

Type I error rate will be controlled over the primary and key secondary outcome using a gatekeeping procedure. Superiority of surgery for percentage predicted VO_2peak_ will only be concluded if superiority is concluded for the SF-36v2 PF score.

Other secondary outcomes will be analysed similarly, using appropriate regression techniques. For all analyses, point estimates, CIs and p values, where appropriate, will be presented.

The impact of missing data will be assessed by examining its extent, and whether it is missing at random (MAR) or is informative. We will consider the use of multiple imputation methods and sensitivity analyses if data are missing to a sufficient extent; in the event of differential missing data rates between arms, sensitivity analyses will be undertaken, including a tipping point analysis that determines how different from MAR the missing data would need to be to change the conclusion of the study.

Outcome data will be analysed at two time points, with the primary effectiveness and cost effectiveness analyses occurring once 1-year data are available for all participants, and further analysis once 3-year follow-up data are available.

### Health economic analysis

The economic evaluation will include a within-trial (to estimate the incremental cost per QALY gained at 1 year) and a model-based economic evaluation (to extrapolate costs and QALYs over a lifetime time horizon). The analyses will take the perspective of the UK NHSs and Personal Social Services. A wider societal perspective will also be considered by analysing the costs borne by the participants and their carers. The analysis will be conducted following the UK National Institute for Health and Care Excellence best practice guidelines.^32^

The within-trial analysis, in the form of a cost-utility analysis, will be conducted on an ITT principle. Costs will include intervention costs derived from a micro-costing exercise conducted at individual study centres. Costs associated with use of health and care services will be calculated from the responses to SUQ completed by participants at 6 months and 1 year follow-up. Unit costs for all healthcare services will be obtained from using routine data sources.^33 34^ These will then be combined with the resource use reported by each participant to derive the mean cost per trial participant.

QALYs will be estimated from responses to the EQ-5D-5L administered at baseline, 6 months and 1 year follow-up. Responses to the EQ-5D-5L will be converted into utility values using recommended scoring algorithms and QALYs estimated using the area under the curve approach and a mean QALY per arm calculated. A sensitivity analysis will estimate QALYs from responses to the SF-36v2 administered at the same time points. The responses to the SF-36v2 will be converted into SF-6D tariffs^35^ to enable us to calculate QALYs using the same method.

We will include stochastic sensitivity analysis, presented as point estimates and cost-effectiveness acceptability curves. We will apply two-stage non-parametric bootstrapping alongside all analyses to minimise any uncertainty surrounding the incremental cost per QALY gained.

Details of the health economics analyses will be set out in the Health Economics Analysis Plan and aligned to the SAP.

### Embedded studies

#### Observational cohort

An additional 100 severely symptomatic PE patients ≥12 years old, in whom it would be unacceptable to wait for 12 months for an intervention, and who have been selected as meeting those criteria by a national multidisciplinary team (MDT),^17^ will be asked to consent to an observational cohort study. People not accepted by the national MDT who may be eligible for the RCT are offered the opportunity to join the RCT at the time of the MDT decision. The observational cohort will run contemporaneously with the RCT. Participants will follow the standard NHS commissioned pathway while undertaking additional CPETs and questionnaires to align with the RCT (Intervention arm) visits. Data from the cohort will be analysed and reported as part of the second planned analysis and will not form part of the primary analysis or primary reporting for the trial.

#### Discrete choice experiment (DCE)

Participants’ preferences for different attributes of the surgical strategies in the trial will be elicited using a DCE. Data collection will take place at approximately 18 months following surgery for each participant. The DCE will present individuals with a series of hypothetical alternative choices, usually pairwise, differing in their attributes and levels, and asking them to indicate their preference. The key attributes will be identified from the literature and discussions with patient groups and other stakeholders. The inclusion of a cost attribute will enable marginal WTP for a unit change in each attribute to be calculated. Appropriate statistical analysis (eg, multinomial logistic regression techniques) will be applied to the data. The results of the DCE will inform the relative importance of attributes and participants’ WTP for the surgical interventions included in the trial. The WTP results will be used to calculate the incremental net benefit of the interventions under the framework of cost-benefit analysis.

### Trial conduct and governance

The Trial Management Group oversees all day-to-day aspects to ensure that the protocol is adhered to, ensuring participant safety and data integrity; they meet approximately monthly. The trial is sponsored by South Tees Hospitals NHS Foundation Trust. The IDMEC report to the TSC provides advice on the ongoing conduct and safety; they meet at least annually. The TSC, where independent members are in the majority, provides overall supervision; they meet at least annually.

### Patient and public involvement

The research team includes two patient co-applicants. These co-applicants had input into the original application for funding, the protocol, patient information sheets and the patient-facing information/videos posted on the trial website. A third patient representative is a member of the TSC who helps provide oversight to the trial.

### Inclusivity

To ensure that RESTORE recruits a representative sample of participants, a number of steps have been included:

All potential participants will have their full postcode included on the study screening logs, to enable the regular monitoring of those that do and do not take part to ensure that participants from across income deciles are participating.

The trial has opened centres across England, including in areas with higher proportions of ethnic minorities, and those with large geographical catchments to ensure we reach across the largest possible population of people with PE.

The research team also works with the charity Pectus Matters and the pectus support UK group on Facebook to engage with patients and carers.

We have included multiple modes of follow-up to avoid unnecessary hospital visits and will enable follow-up (eg, CPET) at more local hospitals, where possible. We have provided a travel budget for research visits. We have translated information sheets and provide a centralised interpretation service for completion of questionnaires as required.

## Ethics and dissemination

The trial is prospectively registered (ISRCTN11359779) and will be conducted in compliance with the principles of the Declaration of Helsinki 2013^36^ and the principles of Good Clinical Practice and in accordance with the UK Framework for Health and Social Care Research. It was approved by the East of Scotland Research and Ethics Service 24/ES/0034, coordinated by the Health Research Authority and Health and Care Research Wales. Participants who are ≥16 years of age will be required to provide written informed consent. Participants <16 years of age who are not judged to be Gillick competent will need to provide written assent, and written informed consent from a parent/guardian will be obtained. The consent process may be performed remotely according to individual trial centre requirements. An example of the consent form for participants aged 16 or over is provided as online supplemental information C.

Results will be published in peer-reviewed journals, presented at conferences and communicated to NHS England and stakeholders.

## Supplementary material

10.1136/bmjopen-2025-113818 online supplemental file 1

10.1136/bmjopen-2025-113818 online supplemental file 2

10.1136/bmjopen-2025-113818 online supplemental file 3
