# Supplementary material for: Surgery versus conservative management for severe pectus excavatum (RESTORE): protocol for a multicentre, randomised, controlled superiority trial
Source: BMJ Open. 2025 Dec 24;15(12):e113818. doi: 10.1136/bmjopen-2025-113818 (PMC12742100; doi:10.1136/bmjopen-2025-113818)
Supplement: online supplemental file 1 [file bmjopen-15-12-s001.docx]

**RESTORE Trial**

**Supplementary information A**

**Documentation of parameters from the Cross-sectional imaging of the chest**

We will collect parameters from the CT thorax, measured BOTH on expiration and inspiration. These are defined below.

1. The Haller Index,

2. The Correction Index,

3. The Vertebral Index,

4. The Depression Index

5. The Asymmetry Index,

6. The Cardiac Compression Index,

7. The cardiac asymmetry Index,

8. The Titanic Index

9. Sternal torsion angle and direction.

**1. The Haller Index (HI)**

The Haller Index is calculated by dividing the inner width of the chest at the widest point (T) by the distance between the posterior surface of the sternum and anterior surface of the spine. The CT scan image selected to make the calculation should be the image with the minimum anteroposterior (APmin) distance between the anterior chest and the spine, even if this image is at the level of the xiphisternum or below. Also, the lowest point does not have to be in the midline if there is asymmetry and the lowest point is to one side of the midline. Normal cut off is 2.5 and the definition of severe is greater than 3.25.

Haller Index formula: = T / APmin

**2. The Correction Index (CI)**

The CI is the expected percentage improvement in the AP distance if repaired (*i.e.*, when the sternum is lifted to make the anterior sternum on a horizontal plane with the ribs).

Measured by subtracting the minimum depth of the chest above the vertebra from the maximum depth of the chest above this line and then expressing this as a percentage (divide by the maximum depth (Max-Min)/Max). A normal Correction Index is <5% and a severe correction index is regarded as >20%. Of note the measurement for the minimum depth of the chest (APmin) is the same vertical measurement used for the denominator of the Haller index. When measuring the maximum AP diameter (APmax), it is defined as the maximum AP diameter so if there is an asymmetrical chest the highest internal point in the chest should be used down to the anterior vertebral line.

Correction Index formula: [(APmax – APmin) / APmax] x 100.

**3. Vertebral Index**

This is an index used as an alternative to the Haller index which is also independent of the width of the chest and uses the depth of the vertebral body (Vap) as compared to the distance between the sternum and anterior vertebral body (APmin) expressed as a percentage. The vertebral body that corresponds to the minimum distance between sternum and vertebral body is measured in the midline. Of note it is just the vertebral body, not the whole vertebra (i.e. not to the posterior spinous process or past the spinal cord). A Vertebral Index of more than 26 indicates severe pectus excavatum.

Vertebral Index formula = [Vap /(Vap + APmin)] x 100

**4. Depression Index**

This is a similar index to the vertebral index in that it uses the vertebral body as a surrogate for the patient’s height in order to provide an index that is related to the size of the participant. This measures the severity of the dip in the chest. The Measurement of the dip in the chest is from a line across the two most anterior ribs (Not soft tissue) and then measured vertically down to the anterior table of the sternum or xiphisternum (Dip), and the transverse diameter of the vertebral body (Vl) are measured. A Depression Index over 0.2 is considered abnormal.

Depression Index formula = Dip / Vl.

**5. Asymmetry Index**

This index is used is to determine the extent that the PE deformity is asymmetrical. The fullest inner depth of the right chest (R) is divided by the fullest inner depth of the left chest (L) and expressed as a percentage.

Asymmetry Index Formula = R / L

**6. The Cardiac Compression Index (CCI - The Heart’s Haller Index)**

This score can be difficult to measure if the heart is fully displaced into the left chest or those with a left sided depression whereby the deepest point of the depression is to the left of the midline. The Transverse diameter is the widest transverse diameter of the full cardiac outline (H). It may be on a different level as was measured for the Haller Index. This full cardiac outline may include the inferior vena cava and with non-contrast scans it can be difficult to determine the exact cardiac border on the right side of the pericardium. Thus, it is acceptable to extend this line to the right to include the IVC if necessary, and the point of measurement is below the atrium. The aim is to measure the minimum AP diameter of the heart in the midline and this is the AP diameter of the cardiac structures in the pericardium (M). This measurement needs to be through the right atrium. If there is no heart in the midline due to leftward cardiac displacement, then the measurement line should be moved to the left until there is a significant portion of the atrium and then it should be used for the measurement of (M). A Cardiac Compression Index of 1.82 or above is considered abnormal.

Cardiac Compression Index formula = H / M

**7. The Cardiac Asymmetry Index**

This is the vertical height of the heart at the greatest point (P) divided by the vertical height under the xiphisternum (M). A Cardiac Asymmetry Index of 1.15 or above is considered abnormal.

Cardiac Asymmetry Index formula = P / M

**8. Titanic Index**

This measurement is a reflection of the height of the defect in the sagittal plane. We define the Titanic Index as the percentage of sunken sternum. Using the heights of the axial image slices, the proportion of sternum that lies behind the anterior costal line can be measured. The upper border of the sternal manubrium is point (A), The point where the sternum begins to sink behind the anterior costal line is point (B), and the xiphisternal joint is point (C).

The Titanic Index formula = [(C-B)/(C-A) x 100]

**9. Sternal torsion**

This is the angle of torsion of the sternum at its maximum torsion. Severe torsion has been defined as more than 30 degrees. This angle is usually taken from the posterior table of the sternum.
