# Supplementary material for: Surgery versus conservative management for severe pectus excavatum (RESTORE): protocol for a multicentre, randomised, controlled superiority trial
Source: BMJ Open. 2025 Dec 24;15(12):e113818. doi: 10.1136/bmjopen-2025-113818 (PMC12742100; doi:10.1136/bmjopen-2025-113818)
Supplement: online supplemental file 2 [file bmjopen-15-12-s002.docx]

RESTORE Trial Protocol

Supplementary information B

This document highlights some of the key requirements for performing CPETs in the RESTORE trial.

Same testing centre

Participants will have the most reproducible results by attending the same CPET centre for all assessments so this is requested per protocol. However, a pragmatic approach will be taken if this cannot happen, and CPETs conducted at sites different to that of baseline will be included in the analysis.

Scheduling

For participants over the age of 16, the standard of care baseline CPET can be performed within a year prior to study consent. If the participant is under the age of 16, then the baseline CPET should be within 6 months prior to consent. If a participant has a CPET older than this, then a repeat test is requested per protocol. The CPET can also be performed after consent but before randomisation.

Valid/sub-optimal testing

We define the following parameters as a valid test:

Respiratory exchange ratio (RER) is greater than 1.1 **OR** heart rate achieved is greater than **85%** of predicted maximum heart rate. If either of these parameters is achieved, we would accept this as being a valid test.

If a suboptimal test is recorded, we request that another test is performed within a 2-month window, but with at least a two-week interval. If a second suboptimal test is recorded, then a 3^rd^ test will not be requested. Sub-optimal data will still be collected (including notation of suboptimal testing). The recommended repetition of a suboptimal test applies to all pre and post-operative tests. We define a suboptimal test as one that has not exercised the participant optimally in order to allow them to achieve their maximal test levels.

Calculation of maximum predicted values

For all ages, we recommend the Tanaka formula 208-(0.7 x age) for calculating max HR.^[[1]](#endnote-2)^ To determine other predicted maximum values for VO_2_ , O_2_ pulse, Ve, power and CPET ramp, sites may choose to use different prediction tools. For children (<16 years), we would recommend the Blanchard score.^[[2]](#endnote-3)^ As participants become older it is acceptable to change the model used, and we would recommend the SHIP equations for those >16 years of age.^[[3]](#endnote-4)^ For study analysis and reporting purposes, the Blanchard and SHIP formulae (according to the participant’s age)^3,4^ will be used.

Pre-test FEV1 and FVC

***Ideally***, all participants would have forced (FEV1 and FVC) measurements taken immediately prior to the CPET using the CPET equipment. Same day measurements would be our next preference. However, if spirometry on the same day is not technically possible, reliably measured values within the preceding 3 months is acceptable. Spirometry should be performed according to ARTP guidance.^[[4]](#endnote-5)^

GPAQ scoring

The patient’s exercise history will be obtained using the Global Physical Activity Questionnaire (GPAQ) and analysis guide on the WHO Website:

<https://www.who.int/publications/m/item/global-physical-activity-questionnaire>

***Ideally*** this questionnaire should be administered in the clinic before CPET. However, the RESTORE trial will allow this to be completed remotely in order to facilitate data capture.

CPET Protocol

The CPET should be performed according to the ARTP guidelines 2021. The use of cycle ergometry is mandated. Treadmill or hand crank may not be used as an alternative. All centres should follow the best practice document published by the ARTP in the BMJ open respiratory journal 2021.^[[5]](#endnote-6)^

# The rest phase

The rest phase should be a minimum period of 3 minutes to allow participants to reach a resting equilibrium. We recommend that resting values be taken during the last 20-30 seconds of the resting period including BP. Automated or manual BP measurements are acceptable.

# Warm up non-loaded phase

This should be 3 minutes of non-loaded cycling.

# The ramp (loaded) phase

A ramp protocol should be used. The ramp protocol should be determined by the SHIP equations^3^ for those 16 and over and Blanchard^2^ formulae for those <16 years, to calculate the optimal ramp incrementation rate. From this predicted ramp calculation, the incremental ramp should be set to the nearest W protocol as per your centre’s equipment specification. For example a predicted 11.6W ramp would be adjusted to 10W if your centre has 10, 15, 20W ramp increments or 12W would also be acceptable if the equipment is able to do so. Decimal places should be rounded up to the nearest whole number if ≥ 0.5, and they should be rounded down to the nearest whole number if ≤ 0.4. Clinical judgement should be used to amend the calculated ramp incrementation rate based on the patient's functional status to achieve 8-12 mins of ramped exercise, which is important to optimise subsequent analysis.^[[6]](#endnote-7)^ At each subsequent time point for a given patient, the ramp slope may be recalculated to adapt to the patient’s needs/physiological changes.

# Anaerobic threshold (AT)

The Anaerobic threshold (AT) is the oxygen uptake (VO_2_) above which arterial lactate first begins to increase systematically during incremental exercise reflecting increased glycolysis. It is a submaximal measure, independent of effort and is expressed in ml/kg/min or ml/min.

Recommended stepwise approach to measuring AT for the RESTORE trial:

- Criterion 1 - Modified V-slope or V-slope technique is recommended as the primary method to identify AT. Ideally this should be supported by ≥ 2 of the supplementary criteria below.
- Criterion 2 – Identify hyperventilation relative to Oxygen.
- Criterion 3 – Exclude hyperventilation relative to CO_2_.
- Criterion 4 – the Respiratory Exchange Ratio (RER) should be ≤ 1.0 at the point where the AT is measured.

Where it is not possible to identify the AT using criterion 1, it is acceptable to use a combination of criteria 2 to 4 as an alternative. A centralised review of the anaerobic thresholds will be undertaken to ensure that all centres are making reliable and reproducible decisions with regard to the anaerobic threshold. All other parameters are objective.

# VO_2_ peak

Peak oxygen uptake (VO_2_peak) is defined as the highest oxygen uptake (VO_2_) attained on an incremental test at end-exercise. As such, it is reflective of the patient’s ‘best effort’ on the CPET but it may not reflect what was potentially achievable for that patient, i.e. it is not necessarily a physiologically maximal end-point (VO_2_ max). VO_2_ peak may be influenced by patient volition or effort, and is expressed in ml/kg/min or ml/min. The average VO_2_ peak should be taken over a 15-30 second period rather than a single peak value.

# Recovery phase

The recovery phase should be a minimum of 3 minutes and can last as long as required to ensure patient safety. End of test BP measurements should be taken as soon as practicable and can be automated or manual.

Measurements collected

| Participant Details | CPET location |
| --- | --- |
|  | Date of CPET |
| Demographics | Age (yrs) |
|  | Height (cm) |
|  | Weight (Kg) |
|  | Blanchard corrected weight (under 16s) |
|  | BMI Kg/m^2^ |
|  | Sex at birth |
| Predicted values | Predicted peak O2 pulse (ml/beat) |
|  | Predicted Peak VO_2_ (ml/kg/min) |
|  | Predicted Max HR (bpm) |
|  | Predicted Max Ve (L/min) |
|  | Predicted Max Power (W) |
|  | Predicted Ramp (W/min) |
| Actual ramp used for test | Actual ramp used (W/min) |
| Spirometry | FEV1 predicted |
|  | FEV1 (L) |
|  | % of FEV1 predicted |
|  | FVC predicted |
|  | FVC (L) |
|  | % of FVC predicted |
|  | FEV1/FVC |
| Resting CPET Data | VO_2_ (ml/min/kg) |
|  | Respiratory Rate (breaths/min) |
|  | Ve (L/min) |
|  | Vt (L) |
|  | HR (bpm) |
|  | SpO_2_ (%) |
|  | RER |
|  | O_2_ pulse (ml/beat) |
|  | BP Systolic mmHg |
|  | BP Diastolic mmHg |
| AT CPET Data | VO_2_ (ml/min/kg) |
|  | Ve/VCO_2_ |
|  | RER |
|  | O_2_ pulse (ml/beat) |
| Peak CPET Data | VO_2_ (ml/min/Kg) |
|  | % predicted VO_2_ (ml/min/Kg) |
|  | Ve(L/min) |
|  | % predicted max Ve |
|  | Ve/VCO_2_ |
|  | RER |
|  | HR (bpm) |
|  | % predicted HR max |
|  | O_2_ pulse (ml/beat) |
|  | % predicted O_2_ pulse |
|  | Respiratory Rate (breaths/min) |
|  | Vt (L) |
|  | Breathing Reserve (L) |
|  | % Breathing Reserve (L) |
|  | SpO_2_ (%) |
|  | mBorg |
|  | Peak Power (W) |
|  | % of predicted Max power |
|  | Total Ramp Ex. Time (MM:SS) (Unloaded + loaded) |
|  | Loaded ramp time |
|  | End BP Systolic mmHg |
|  | End BP Diastolic mmHg |
| Was the test stopped early | Yes/No |
|  | Reason for Stopping early:   - Fatigue - Chest pain - Musculoskeletal Pain - SOB - Dizziness - Max HR exceeded - ST depression - ST elevation - BP over limit - BP fail - Arrhythmia - Claudication |
| Were there ECG changes | Yes/No |
| Ex. ECG changes | SVT  PVC  VT  PAC  Heartblock  ST elevation  ST depression  T wave inversion  Bigeminy  Trigeminy  VF |

# REFERENCES

1. Tanaka H, Monahan KD, Seals DR. Age-predicted maximal heart rate revisited. J Am Coll Cardiol. 2001 Jan;37(1):153-6. doi: 10.1016/s0735-1097(00)01054-8. PMID: 11153730. [↑](#endnote-ref-2)
2. Blanchard J, Blais S, Chetaille P, Bisson M, Counil FP, Huard-Girard T, Berbari J, Boulay P, Dallaire F. New Reference Values for Cardiopulmonary Exercise Testing in Children. Med Sci Sports Exerc. 2018 Jun;50(6):1125-1133. doi: 10.1249/MSS.0000000000001559. Erratum in: Med Sci Sports Exerc. 2019 Jul;51(7):1571. PMID: 29346167; PMCID: PMC6023574. [↑](#endnote-ref-3)
3. Reference values for cardiopulmonary exercise testing in healthy volunteers: the SHIP study

   B. Koch, C. Schäper, T. Ittermann, T. Spielhagen, M. Dörr, H. Völzke, C. F. Opitz, R. Ewert, S. Gläser European Respiratory Journal Feb 2009, 33 (2) 389-397; DOI: 10.1183/09031936.00074208. [↑](#endnote-ref-4)
4. Sylvester KP, Clayton N, Cliff I, et al ARTP statement on pulmonary function testing 2020 BMJ Open Respiratory Research 2020;7:e000575. doi: 10.1136/bmjresp-2020-000575 [↑](#endnote-ref-5)
5. Pritchard A, Burns P, Correia J, et al ARTP statement on cardiopulmonary exercise testing 2021 BMJ Open Respiratory Research 2021;8:e001121. doi: 10.1136/bmjresp-2021-001121 [↑](#endnote-ref-6)
6. Buchfuhrer MJ, Hansen JE, Robinson TE, Sue DY, Wasserman K, Whipp BJ. Optimizing the exercise protocol for cardiopulmonary assessment. J Appl Physiol 1983; 55:1558–1564. [↑](#endnote-ref-7)
