# Supplementary material for: Surgery versus conservative management for severe pectus excavatum (RESTORE): protocol for a multicentre, randomised, controlled superiority trial
Source: BMJ Open. 2025 Dec 24;15(12):e113818. doi: 10.1136/bmjopen-2025-113818 (PMC12742100; doi:10.1136/bmjopen-2025-113818)
Supplement: online supplemental file 3 [file bmjopen-15-12-s003.docx]

**RESTORE - TRIAL CONSENT FORM**

| IRAS ID: | 331910 |
| --- | --- |
| Centre No. |  |
| Study Title: | A randomised trial of surgery versus no treatment to RESTORE cardiopulmonary function in severe pectus excavatum |
| Study Doctor: | Full name of principal investigator, title,  institutional affiliation, address, phone number |
| Study Sponsor & Data Controller: | *South Tees Hospitals NHS Foundation Trust* |
| Participant ID: |  |

|  |  | | | Please initial box | |
| --- | --- | --- | --- | --- | --- |
| 1. | I confirm that I have read the Participant Information Sheet for the Randomised Trial dated.................... (version...........) for the above study. I have had the opportunity to consider the information, ask questions and have had these answered satisfactorily. | | |  | |
| 2. | I understand that my participation is voluntary and that I am free to withdraw at any time without giving any reason, without my medical care or legal rights being affected. | | |  | |
| 3. | I understand that relevant sections of my medical notes, including scans, and data collected during the study, may be looked at by individuals from the Sponsor, research team, or from the NHS Trust, where it is relevant to my taking part in this research. I give permission for these individuals to have access to my records. | | |  | |
| 4. | I understand that the information collected about me will be used to support other research in the future, and may be shared as coded data with other researchers. | | |  | |
| 5. | I agree to my General Practitioner being informed of my involvement in the study, and to any necessary exchange of information about me between my GP and the research team. | | |  | |
| 6. | I understand that the information held and maintained by the research team may be used to help contact me or provide information about my health status. | | |  | |
| 7. | I agree that my personal information can be used in the way described in the Participant Information Sheet for the study. | | |  | |
| 8. | I agree to take part in the randomised-controlled trial, where I agree to be allocated by chance to either having early surgery, or, surgery with a delay of one year. | | |  | |
| 9. | I agree to take part in the longer-term follow-up, if this gets funded. | | | YES | NO |
| 10. | I agree to my scans being collected for future research. | | | YES | NO |
|  | | | | | |
| Name of Participant: | | Date: | Signature: | | |
| Name of Person seeking consent: | | Date: | Signature: | | |
